# The Medication of the Mind

**Published:** 1889-11-02

**Authors:** 


### The Hospital

A WEEKLY INSTITUTIONAL JOURNAL OF

**Science, Medicine, Nursing, and Philanthropy.**

*For Hospitals, Asylums, Medical Practitioners, Students, Nurses, Families, Parishes, Congregations, and the Charitable Public.*

VOL. VII. No. 162.] [NOVEMBER 2, 1889.

### THE MEDICATION OF THE MIND.

FOR the purposes of the physician man must be regarded as a material organism, with mental and spiritual functions. There is not here necessarily implied any conflict with the metaphysician or the theologian, each of whom is justified by his interpretation of facts in dividing man into body and mind, or into body, soul, and spirit. But the physician who studies man with modern and scientific eyes and temper learns to regard him for all the practical purposes of his art not as three, or even two more or less distinct entities, described by the several terms "body," "soul," and "spirit," but as a single organism, "one and indivisible."

Those medical workers to whom the facts of science are living and present realities, and not merely the means of academical pastime, habitually regard man in relation to his environment. It is indeed impossible for the philosophic mind to consider any living organism except in conjunction with all the surrounding circumstances of time, place, and condition, which belong to it in its natural state. Environment, natural environment, is, philosphically considered, an integral part of the organism. That is to say, it is that without which the organism could not be what it is. Very familiar examples may convince us of this in a moment. The Scottish Highlander, with his long tawny beard, his firm-set features, his expression of concentrated resolution, his immovable obstinacy of temperament—whence and why is he what he so constantly is? A stern mountainous region, thin and rocky soil, a pitiless climate, and a stormy ocean have been his lifelong and unfriendly environment for countless generations. If he was to survive at all, it could only be by the exercise of a resolution sterner than the sternest of his mountains, and a courage more invincible than the fury of the pitiless blast or the rage of the tempestuous northern sea. He has survived; and when hundreds of his kind are gathered together into a drilled regiment of fighting men it needs no philosopher to discover that permanent and exceptional conditions have operated to the production of creatures so masculine and so terrible. Burns drew but little on his imagination when picturing to himself the march past of a thousand such fighting men, he felt "the trembling earth resound their tread." At the opposite end of the scale is the soft-skinned, silky-haired, meditative, but nerveless and feeble-willed Asiatic of the Hindoo type. A milder sky, a more productive soil, ages of existence which have called forth no continuous concentrated effort and resolve, have made him also what he is, and they are, so to speak, an essential part of himself.

If then it be undoubted that to consider a man apart from his environment is not to consider him at all in any complete sense, is it not incumbent upon every scientific physician to have every man's environment present in his mind, as definitely and actually as the man himself is present in the consulting-room? This patient's dyspepsia must be considered in conjunction with his natural constitution, his school life, the bad air of the locality in which he lives, the stuffy or draughty office in which he works, the temper of his wife, the worry of his children, his intellectual characteristics, his moral stand-point. That woman's bloodlessness and general prostration must be regarded in relation to her indoor life, her small appetite, her poor digestion, her disturbed sleep, her limited means, her want of governing capacity in the management of house and servants, her family history, and all that appertains to her. These are the commonplaces of daily experience. Nobody denies them.

We contend that at the present time there is, and for the last half-century there has been evolving an altered relation between body and mind. The mind—the brain, in short—of the present generation is more generally and more intensely active than was the mind of immediately preceding generations. This is not the same as saying that the average man of the present generation has more sense and judgment than his grandfather, or that the poets and philosophers of the present age are greater than Shakespeare or Goethe, than Descartes or Newton. It is only affirming that the average man's mind is much more active and is subjected to much more wear and tear than was the average man's mind of the sixteenth, seventeenth, and eighteenth centuries. And it is, therefore, imperatively incumbent upon the practical physician that he constantly study, understand, and practise the "medication of the mind." In the consideration of almost every individual case it is as necessary to take into the "brief" the state of the mind as it is to include the condition of the teeth, or the stomach, or the bowels, or any other primary organ or function of the body.

But how is the mind to be dealt with in any practical way by the ordinary physician? He must use the same methods and proceed on the same lines as he does in his treatment of the body. If the liver is congested, a cholagogue and an aperient will relieve the congestion, and an acid tonic will bring back cheerful health. So, if the mind be congested by too great a burden of crude ideas, and of probably causeless cares, is it too much to ask of the doctor that he shall understand the case, that he shall firmly and authoritatively make known his judgment to the patient, and that he shall administer, with the same confidence as he administers his blue-pill and his effervescing draught, a dose of clear, plain sense to remove the crudities and to clear away the anxieties of the mind? Every physician who deserves that honoured name establishes in his patient's mind a just and reasonable confidence; and it is quite certain that an honest appeal to that confidence has ordinarily as real and beneficial an effect on the patient's judgment as the cholagogue and the effervescing draught have on his stomach and his liver.

There are certain great mental crises in the history of most men in which the physician can apparently be of little or no service. But are there no physical crises where a sympathetic and intelligent watchfulness is almost the only service that can be rendered to the patient? What, for example, can be done in a severe case of typhoid fever except to watch, and, when possible, moderate the severest symptoms, to help the strength by such food as may be safely taken, to remove all external sources of depression and danger, and to await the issue of the deadly struggle with patience and with hope? But these services, small and inconsequent as they seem, in many cases make all the difference between a fatal and a victorious termination of the strife. Many a life is undoubtedly saved by just such small services as these. There is the closest analogy between a physical crisis of this kind and the overwhelming sorrow that rushes down upon a man in the loss of wife, or parent, or child, or in the ruin and destruction of his fortune. The mind collapses, and the body is prostrated with it. Can any metaphysician separate the two at moments such as these? What can be done by the physician? Little, and yet much! Exactly as in the case of the typhoid fever, he can watch intelligently. He can give the soothing draught, he can gently insist upon the suitable food, he can at the right moment bring friends or children on the scene, he can suggest duty, Divine consolation, the "medicine of work," he can have the wearied body and the prostrate mind taken away from the heart-breaking environment and encompassed by sights and sounds that dispose to rest, to soothing, and to hope. Thus he stands, like the spirit of gracious wisdom, between the crushed soul and utter despair.

In times, like the present, of extraordinary mental activity, and consequent wear and tear and injury, not to know how to "minister to a mind diseased" is to be ignorant of half the practical part of a physician's daily duty. Yet many completely fail here, and that for reasons which are not far to seek. A defective early education in the literature of the household, and a crude, ignorant, and unphilosophical materialism which is prevalent in many of the medical schools, are two of the main causes of the physician's helplessness in this department of his work. If to this be added a natural defect of sympathy on the part of many young men that prompts them to seek a calling in which they think physical science plays the principal part, the chief causes of failure will have been pointed out. These faults are all remediable, and it is quite certain that they must be remedied if medicine is to do what it can and what it ought for present and future generations. A wider general culture and a deeper sympathy with all that belongs to man as man are among the imperative requirements of him who aspires to do in his own generation the noble and incomparable work of the true physician.

